# Development of the nursing associate professional identity: A longitudinal qualitative study

**DOI:** 10.1002/nop2.2131

**Published:** 2024-03-07

**Authors:** Rachel King, Sara Laker, Bethany Taylor, Tony Ryan, Emily Wood, Angela Tod, Michaela Senek, Sally Snowden, Steve Robertson

**Affiliations:** ^1^ The School of Allied Health Professions, Nursing and Midwifery The University of Sheffield Sheffield UK; ^2^ Winona State University ‐ College of Nursing and Health Sciences Winona Minnesota USA; ^3^ Sheffield Centre for Health and Related Research (SCHARR), School of Medicine and Population Health The University of Sheffield Sheffield UK

**Keywords:** health professions, longitudinal study, nursing associates, professional identity formation

## Abstract

**Aim:**

The aim of this study was to understand the factors that contribute to the development of the nursing associate professional identity.

**Design:**

A 3‐year longitudinal qualitative study of trainee nursing associates.

**Methods:**

Trainee nursing associates in England were interviewed remotely annually in February 2020, March 2021 and March 2022. They also provided diary entries. Data were anonymised, transcribed and analysed thematically.

**Results:**

Nursing associate professional identity was developed through: increased knowledge, skills and responsibility; and self‐perceptions of identity alongside responses to the role by colleagues. Tensions arose when the scope of practice expected by organisations differed from that expected by the nursing associates. Frustrations occurred when nursing associates were perceived as substitutes for Registered Nurses in the context of nursing workforce shortages.

**Conclusion:**

Nursing associates in this study clearly valued their new knowledge, skills and responsibility, enabling them to provide enhanced patient care. Increased clarity of role boundaries is necessary in enhancing the professional identity of nursing associates and reducing inter‐professional tensions arising from role ambiguity within health and social care organisations.

**Implications for the Profession:**

National guidance and employers should provide clarity on the boundaries of the nursing associate role which will strengthen their professional identity and mitigate role ambiguity within health and social care organisations.

**Reporting Method:**

The Consolidated Criteria for Reporting Qualitative Research has been used to guide reporting.

**Patient of Public Contribution:**

A patient and public involvement group was consulted during the initial study design stage.

**Impact:**

- This study aimed to understand the factors which contribute to the development of a nursing associate professional identity.
- Nursing associate professional identity is developed through increased knowledge, skills and responsibility, and the perceptions of identity by participants themselves and their colleagues.
- The findings should inform the implementation of initiatives to clarify nursing associate role boundaries and the development of similar roles internationally.

## 1 INTRODUCTION

The nursing associate (NA) role has recently been introduced in England as a bridge between the Registered Nurse (RN) and healthcare assistant (HCA) roles. When similar roles have been implemented in other parts of the world, both NAs themselves and their colleagues have experienced confusion around the boundaries of their scope of practice. This study aims to understand how the professional identity of NAs is developed, using a longitudinal qualitative design.

## 2 BACKGROUND

The Shape of Caring review (HEE, 2015) of healthcare assistant (HCA) and Registered Nurse (RN) work and training identified a perceived gap between these two well‐established roles. It recommended the introduction of a second‐level nursing role, the nursing associate (NA), as a bridge between the two roles. This follows the phasing out of a previous second‐level nursing role in the United Kingdom, the Enrolled Nurse, in the 1990s as part of a move to an all‐graduate‐level nursing profession (Glasper, 2016). Important in this move was a transfer of nurse education and training in the United Kingdom away from hospital settings and into Higher Education Institution settings. This shifted the dynamics of the vocational nature of training. Nurse students were no longer included in staffing numbers in clinical settings and became seen as more removed from the practical aspects of learning even though placement hours remained nationally mandated (NMC, 2018a). The move to reinstate a second‐level nursing role in England aligns with similar workforce models found in other high‐income countries such as Australia, New Zealand and North America (Lucas et al., 2021; The Health Foundation et al., 2018). In general, second‐level nurses undertake a 2‐year diploma‐level programme and work under the supervision of RNs (who study for a 3‐year degree), and this approach was replicated in the implementation of the NA programme (Lucas et al., 2021). In the United Kingdom, theory and clinical skills training are delivered through universities and clinical settings. However, the training of NAs differs from that of RNs. NA trainees are primarily full‐time health service employees based in a clinical area, with limited time release for university study and alternative placements. Conversely, RN trainees are primarily university students who are provided with clinical placements. As mentioned previously, the standards of proficiency and programme content emphasis for NAs is on generic skills that can enable them to meet the needs of anyone they encounter regardless of life stage or health conditions (Nursing and Midwifery Council, 2018b).

One of the drivers for the development of the NA role was to provide career development for HCAs, a group who otherwise lacked such opportunities (HEE, 2015; King et al., 2020). It also provided a new route into RN training, at a time when the profession was, and still is experiencing significant workforce shortages with difficulties in recruitment and retention (WHO, 2022).

Health Education England (2017) has set out a framework for the knowledge and skills that NAs will develop over their programme of study. NAs are trained to undertake clinical skills similar to RNs, for example, cannulation, ECGs, catheterisation and administration of medication including intramuscular and subcutaneous injection (NMC, 2018b). On qualification, NAs can undertake additional training to administer intravenous drugs and immunisations and undertake cervical screening (RCN, 2023). Although there is some overlap with RN competencies, attempts to define role boundaries have been made by the Nursing and Midwifery Council (2019) (Box 1). Both are accountable professionals, responsible for promoting health, providing care, working in teams and improving the safety and quality of patient care. The key differences (in bold italics) are that RNs hold a leadership role and are responsible for assessing needs, coordinating, planning and evaluating care.

BOX 1 **Main differences between the NA and RN roles**

**Table:** 

Nursing associate (NA)	Registered Nurse (RN)
Be an accountable professional	Be an accountable professional
Promoting health and preventing ill health	Promoting health and preventing ill health
Provide and *monitor* care	Provide and *evaluate* care
Working in teams	*Leading and managing nursing care* and working in teams
Improving safety and quality of care	Improving safety and quality of care
*Contributing to integrated* care	*Coordinating* care
	*Assessing needs and planning care*

(Nursing and Midwifery Council, 2019).

Although NAs work alongside members of the nursing profession, they do not hold the title of ‘registered nurse’, creating challenges in relation to the formation and maintenance of ‘registered nursing associates’ identity. Professions are constructed by identifying problems, devising strategies to solve them and adopting that work as their own (Abbott, 2010). They are defined as having ownership of particular areas of knowledge, expertise and decision‐making (Traynor et al., 2010).

In distinguishing one profession from another, professional identity is formed through professional socialisation in both academic and clinical settings (Maginnis, 2018). Maginnis (2018) emphasises the importance of role modelling in building a nursing professional identity. Similarly, in a study of student nurses, clinical experience, role modelling, critical thinking and reflection were identified as important factors in building professional identity (Fitzgerald & Clukey, 2021).

In a concept analysis of professional identity, Fitzgerald (2020) identified the following key attributes: skills, knowledge, values and ethics; personal identity; group identity; and the influence of the context of care. All of these elements of professional identity begin to develop during the training process (Fitzgerald, 2020) and are important to consider in workforce retention strategies as a lack of professional identity may result in insecurity and a feeling of dislocation from the profession (Maginnis, 2018).

Workforce boundaries can blur when work is taken on by another profession through substitution (Nancarrow & Borthwick, 2005). Boundary blurring and subsequent knowledge sharing can enable less powerful or emerging professions to advance their positions (Currie & White, 2012). Much research on boundary blurring has explored the interface between nursing and medicine (Allen, 1997; Johannessen, 2018; Nancarrow & Borthwick, 2005; Schober et al., 2016) and more recently between the physicians' associate role and medicine (Halter et al., 2018). In the case of the new NA role, it is important to explore whether role distinctions set out by the regulator are clear in practice, or whether boundary blurring occurs with RN‐specific competencies and roles. It is also important to consider the role that the more vocationally embedded nature of NA training plays in its identity construction. In countries where second‐level nursing roles have had more time to become embedded into healthcare teams, studies have identified role confusion, with blurring between the scope of practice of RNs and LPNs, for example, in Finland (Lavander et al., 2017) and Canada (Lankshear et al., 2016).

Threats to professional identity can be fuelled by discrepancies between the ideals and realities of the scope of practice (Fitzgerald, 2020). An international review of the perspectives of healthcare professionals on second‐level nurses found that some are devalued, while others are given increased responsibility, leading to boundary confusion (Lucas et al., 2021). A culture shift in valuing the care provided by second‐level nursing is recommended, along with a system‐level increase in role clarity within healthcare teams (Lucas et al., 2021).

Previous studies on NAs have focused on the experiences of trainees (King et al., 2020; Robertson et al., 2021) and the integration of newly qualified NAs into the workplace (Kessler et al., 2020). This paper explores the issues associated with forming a distinct professional identity in the NA role as they occupy the space between HCAs and RNs.

## 3 THE STUDY

### 3.1 Aim

The aim of this study was to understand the factors that contribute to the development of the nursing associate's professional identity.

## 4 METHODS

### 4.1 Design

A 3‐year longitudinal qualitative study of trainee NAs in England using interviews and diary entries.

### 4.2 Sampling and recruitment

Trainees were recruited from a larger cohort study of NAs which commenced in 2019; the results of which are published elsewhere *[authors]*. Longitudinal data provided valuable insight into the shifting experiences and perspectives of participants in developing their professional identity as they moved through training and into the NA role (Neale, 2020). The Consolidated Criteria for Reporting Qualitative Research (COREQ) has been used to guide reporting (Tong et al., 2007).

After volunteering to be contacted for interview via a survey (Robertson et al., 2021), trainee NAs were purposively sampled to ensure diversity by geographical region, gender, age and ethnicity. Initially, 20 trainees were invited to participate by emailing a participant information sheet and consent form, and 5 more were invited to join the longitudinal study in year 2. A total of 14 trainees from across England agreed to take part in year 1; 17 agreed in year 2 (four new and one from year 1 did not respond) and 12 agreed in year 3 (one declined and five did not respond). Following recruitment, each trainee NA was given a numerical code (TNA 1 to TNA 18) (although they became qualified NAs during data collection, these codes were used throughout to ensure consistency). It is likely that the excellent retention rate across the study (with 10 participants volunteering to be interviewed on all three occasions) was aided by the careful development of rapport in year 1 (Hermanowicz, 2013).

### 4.3 Data abstraction

Semi‐structured interviews were undertaken in February 2020, March 2021 and March 2022, and diary data were collected via Google Forms throughout the 3‐year study. The longitudinal design allowed the interviewers flexibility to improvise and explore key issues during the natural flow of conversation. Interviews were arranged via email and undertaken over telephone or video call (Zoom or Google Meet) at a time convenient to participants. A total of 43 interviews took place over the 3‐year period. They lasted between 21 and 50 min (average 33 min). Data collection was undertaken by four researchers (RK, SR, BT, SL) (one male and three females), three are Registered Nurses and one is not a registered healthcare professional. All have extensive post‐doctoral experience in undertaking qualitative healthcare research.

The interview topic guide (see Box 2) was developed from key issues identified in policy documents and during previous preliminary research (King et al., 2020) and piloted with two trainees to ensure clarity of the questions. The year 2 and 3 interviews explored the same broad themes as the first year of interviews in addition to revisiting and updating previous understanding in an iterative way (Neale, 2020). It is important to follow NAs during their training and into qualification as this is a crucial time in developing professional identity (Fitzgerald, 2020). Interviews were audio‐recorded using an encrypted device, transcribed and anonymised prior to data analysis. Participants were offered a £10 shopping voucher following each interview as a thank you for participating.

BOX 2 **Interview topic guide**

**Motivations**

Where did you work prior to starting NA training?

What made you decide to undertake NA training?

**Experiences**

How are you finding the TNA role? Has it met your expectations? *In what way(s)*?

How does the TNA/NA role differ from your previous roles?

How are you finding the training?

What are you enjoying most about the TNA/NA role?

Are there things you are finding difficult? *What are these*?

Can you describe your support networks?

**Identity**

How do patients and your colleagues respond to your role?

How do you think your role fits in the wider healthcare team?

**Career aspirations**

What are your career plans over the next 4–5 years? *What has influenced that*?

Participants were emailed a diary prompt every 3 months over a 3‐year period with a link to an electronic Google Form which collected entries (see Box 3). A total of 20 diary entries were completed between Feb 2020 and May 2022 by five participants. Previous research has found that diaries are useful in capturing data about workplace identity (Radcliffe, 2013).

BOX 3 **Diary prompt**

We would like you to make notes of any experiences that stand out to you as important during your training/work, particularly those that make you reflect on your motivations for becoming a nursing associate, or that alter how you think about your career path. These experiences could be big or small, could be a single event or take place over a longer period.

### 4.4 Data analysis

We engaged with Braun and Clarke's (2020) six‐step framework for reflexive thematic analysis; data familiarisation; systematic data coding; generating initial themes; developing and reviewing themes; refining and naming themes; and writing the report, while attending to the dynamic nature of longitudinal qualitative data (Neale, 2020). In managing the data, we used Quirkos© (version 2.4.1) computer‐assisted qualitative data analysis software (CAQDAS). Data relating to identity formation were initially extracted and coded by *[author initials]*, then contributed to further refining codes, categories and to the developing and naming of themes.

### 4.5 Ethical considerations

Written informed consent was gained prior to collecting interview and diary data from participants. They were sent an email annually, inviting them to the next interview and reminding them that they could withdraw at any time, although anonymised data incorporated in the analysis would not be removed. Research Ethics Committee approval was gained from the University Research Ethics Committee [Ref: 026355].

### 4.6 Rigour

Rigour was enhanced using several strategies. A detailed description of the study design was provided and included both researcher and source triangulation (collecting both interview and diary data) (Ritchie et al., 2014). Furthermore, we assured trustworthiness by attending to discrepancies in the data through collaborative team discussion. Pragmatic data saturation was reached through the generation and contextualisation of categories (Low, 2019).

## 5 FINDINGS

### 5.1 Participants

The demographics of participants are presented in Table 1.

**TABLE 1:** Participant demographics.

Number of participants	Year 1	Year 2	Year 3
	14	17^a^	12^b^
Age range			
20–29	3	4	2
30–39	4	6	5
40–49	4	5	3
50–59	3	2	2
Gender			
Male	3	3	3
Female	11	14	9
Ethnicity			
White British	10	13	11
Black British	1	1	0
Black (non‐UK)	2	2	1
Asian British	1	1	0

^a^ 4 new recruits and 1 previous participant from year 1 did not respond.

^b^ 10 participants were from across all 3 years and 2 participants from year 2.

Data analysis provided two key themes relating to identity formation; firstly, through the acquisition of skills, knowledge and a greater sense of responsibility, and secondly, in relation to how the role is understood by NAs and their colleagues (Table 2). Extracts from interview and diary data are used to illustrate these themes, with examples provided of how perspectives and experiences have developed over time.

**TABLE 2:** Themes and sub‐themes.

Themes	Sub‐themes
1 Development of knowledge, skills and responsibility	Development of knowledge and skills
	Increased sense of responsibility
2 Perceptions of identity	How the role is understood by NAs
	Colleagues' responses to the NA role
	Strategies to improve role clarity

### 5.2 Theme 1: Development of knowledge, skills and responsibility

#### 5.2.1 Development of knowledge and skills

During training, NAs developed a broad range of knowledge and skills to meet the needs of their new role. Participants valued the opportunity for professional development and felt able to provide a greater level of evidence‐based care than when they worked as HCAs; university learning enhanced their theoretical understanding and how this linked to vocational tasks. Some learning developed new competencies. One participant explained how the knowledge and skills acquired across the four fields of nursing enabled them to provide better physical healthcare for people in a mental health setting:

I've started doing shifts in mental health over the last month and I've picked up a lot of knowledge [through training] about physical health so, I'm finding I can delegate a lot more confidently than I would previously and, picking up if somebody's got this observation then, you know, it could be this, this and this. And I know what questions to ask because of what I've learnt. TNA9 (yr 3)

Other participants talked about their enthusiasm for university learning and how this has improved their work through increased knowledge. For example, TNA 11 demonstrates growth over time in their appreciation of evidence‐based practice; from the value of assessments to a broader view of understanding the evidence behind holistic care:

I've learnt so much from doing this, even essays that you think are pointless or you roll your eyes with, it's the terminology, the communication side of it. Every time I've done an essay or something, I've learnt so much. TNA11 (yr 2)

You have to look at the whole picture, you're not just treating [name's] broken leg, you're having a holistic approach to her and things like that. I love just the background to everything, everything you do, there's a reason why. TNA11 (yr 3)

Where I've always done stuff in patients' best interests, it's understanding the theory behind that a lot more and, you know…why we do what dressings or why we do what behaviour management we apply, it's not a case of because it's always been done, it's the research and understanding behind it. TNA13 (yr 1)

There was a change in attitude towards learning among some participants. For example, for one trainee, some of the academic content of training was perceived as irrelevant but, over time, they reflected on the benefits of developing evidence‐based knowledge and the confidence this brought:

There was a lot of government policy and a lot of stuff that I don't feel that I use now in my role. And I think if we'd had more medicines management and some more anatomy and physiology it definitely would have been a lot better. TNA17 (yr2)

I could underpin what I'm doing more now, whereas as a HCA I probably didn't. But I can look to research now as to why we're doing what we're doing, and I feel more confident since doing my nursing associate [training] to do the things that I do. TNA17 (yr3)

Increased knowledge led to increased confidence in clinical decision‐making, and enhanced care for patients. This is illustrated in the following diary entry:

I love learning new things but more importantly ‘why we do what we do’. The theory behind the practice. My confidence in my skills and ability has increased greatly TNA12 (diary)

The university elements of training often improved care by enhancing the knowledge of what lay behind the vocational tasks that had often been carried out for years by trainees in HCA roles. This improved care was complemented by changes in attitude as they further developed their identities through training.

#### 5.2.2 Increased sense of responsibility

In addition to the impact of new knowledge and skills, participants described the difference that NA training has made in how they approached patient care compared to when they worked as HCAs, for example, by promoting shared decision‐making and person‐centred care:

I think it's just a really, really amazing opportunity to upskill and learn new things… being able to be person‐centred in your caring. Being a bit more useful in the process because as a HCA you're limited to the things you can do and as a NA you can do a little bit extra and help out more and you're more involved in patient care and empowering your patients and helping them to make decisions. TNA1 (yr3)

There was a new passion among some participants to promote learning and development across different professions; valuing opportunities for inter‐professional knowledge sharing and developing an identity more confident with such interactions. The following participant revealed in their first interview a new appreciation for the theoretical underpinning of clinical skills, and in their third interview, they described how they have a role in applying their new knowledge to supporting others:

You know, we can go and do ECGs, take blood, you know, so if there's a list of things that the patient needs, the NA could do that. Although HCAs in many places, such as A&E or the emergency floor, they do all of that anyway, but do they know why? TNA4 (yr 1)

We have doctors visiting our department, medical students, and I just went straight up to them and said, oh have you got any learning outcomes for today, is there anything specific that you need to look at? Just trying to make them feel welcome, and just informed as much as possible. TNA4 (yr 3)

Professional regulation with the Nursing and Midwifery Council (NMC), on qualifying as an NA, was another factor which promoted a feeling of responsibility for their own learning among participants. This increased vocational accountability contributed to their shifting and emerging professional identity.

Everybody's accountable, but when we have got that pin [NMC registration], we are even more so accountable. You've just got to think in a different way, you've really got to step‐up and become more professional about what you're doing. You've got to learn to question things, I've always been like that anyway…So, if I don't know something, I would look it up, but now it's just, kind of, really drummed into you that everything has got to be evidence‐based, which is how it should be. TNA12 (yr 1)

Enhanced knowledge and skills combined with improved confidence and accountability work together in the development of the NA identity. However, developing a new professional identity is not straightforward for the individual, or for others in the clinical settings where training occurs.

### 5.3 Theme 2: Perceptions of role identity

The development of NA professional identity is not only influenced by enhanced knowledge, skills and increased sense of responsibility during training and early career experiences. It is also shaped by how NAs perceive themselves, their role and how they experience the perceptions of others around them in the workplace.

#### 5.3.1 How the role is understood by NAs

Participants in this study were among the first trainee NAs in England, and as a consequence lacked role models in the workplace. This, alongside delays in the development of national guidance on the role, led to some confusion regarding their scope of practice and their role identity. Some were unsure of what the role would entail when they started the programme, with several describing it as similar to the previous UK state Enrolled Nurse (SEN); a role that also sat between HCAs and RNs:

I went home and read what an NA is and, from what I could gather, it's almost like the old SEN role, that was my, sort of vision of it. TNA6 (yr 1)

We're, like a bridge, you know, the usual line, of bridging a gap between HCAs and RNs. And sometimes you talk about the SENs and how it's quite similar to that role and just that we've been to university and we've done our two years of nurse training. TNA9 (yr2)

One participant was more confident of their place in the healthcare team at the start of the study during their first interview. However, this confidence in their professional identity diminished over time.

So, like, the medicine round takes the nurse hours, even for the NA to be able to take over that to enable the nurse, you know, to go on to more complex things is…I think will really benefit the ward. TNA11 (yr 1)

I don't really feel I have a place, I don't really feel I've got an identity at the minute. TNA11 (yr2)

I just say, my role is similar to the old enrolled nurse and I'm just a bridge between them both, it's just in between. TNA11 (yr3)

Some trainees said that they were well placed to enable RNs to undertake more complex care, in light of nursing workforce shortages, by undertaking key tasks:

It's going to free up nurses [RNs] to do other stuff, to concentrate, because they have a lot to be dealing with and the buck stops with them, and there's just not enough nurses and not enough people to help. So, having that in‐between‐person between the HCA and the RN is going to help a lot because the HCAs will come to the RN a lot, ‘Oh, this patient's drip's sounding because they've got an occlusion’. ‘Oh, this person really needs their pain relief’. So, all that stuff that the HCA can't do that only the RN can do, then there's going to be a middleman that's going to be able to help out with that. TNA1 (yr 1)

There was some scepticism among participants about whether the role would meet initial policy objectives or whether it would be dependent on the needs of the organisation. For example, one participant voiced these concerns in the first interview and then described their experiences, which supported their initial concerns in a subsequent interview.

I hope it's going to be what the idea was set out to do and bridge the gap and improve patient care. But I think it depends on what kind of ward you are on and what the staffing is. I think, a lot of feedback I've had from people on the course is that they are just used wherever they can fit in… TNA3 (yr 1)

I feel like we were sold the dream essentially, we were going to be there to support RNs, but I've actually found we are the nurse…I suppose when we first took on the role, when it first came out and a lot of the older nurses said, oh you're cheap nurses blah, blah, and we obviously fiercely defended ourselves, like, ‘we're there for your support’. But actually, being in the role, they've got it right, we are being used to fill in the gaps for band 5s [junior RN grade]. TNA3 (yr3)

Such ambiguity was compounded by the nature of the training and the fact that most were still employed as HCAs with time release for university and alternative placements. This meant that when clinical areas became busy (e.g., during COVID‐19 but not limited to this), training and learning often took second place to vocational demands:

So I was fighting to get my role recognised, and all I was getting was, ‘you're being paid to be here, you're being paid’. So when they were short, I was being chucked in the [HCA] numbers, which is absolutely fine, but not every day. Because I was like, I'm just going to be a really expensive HCA with a PIN, at the end of my training. TNA 3 (Yr2)

You start off the day where you should be but then you just know that during the day it's going to change and you're just going to end up supporting the HCAs again, …I just think even though you know I've got that professional identity they don't still take that into account. TNA15 (Yr2)

Some had become disillusioned by the organisational expectation to work beyond the perceived boundaries of the NA role, as substitutes for RN staff. There was a realisation among some participants that the scope of the NA role was similar to the RN role, leading to a desire to undertake additional training to become an RN.

‘We all feel that we do the job anyway, we do the job as the [band] five, so why not get paid as a band five [junior RN grade].’ TNA11 (yr3)

‘We're told that you can gain any competencies that may be used in your work area. It seems that the only difference will be that we can't take control of a shift. I find this difficult to understand and wonder if we will just be a cheaper version of band five nurses’. TNA5 (diary)

Others had a broader view of the changing landscape of the healthcare workforce, recognising the increased level of decision‐making required by both RNs and advanced practitioners, which creates a space for the new NA role:

The level at which the RNs are coming out at now, they're expected to look after their more acute patients and they're almost working to junior doctor level. So the NAs, those that don't have any desire to continue their journey and they're happy to work as a NA, I think they've got a very firm place in the organisation. TNA14 (yr 1)

Some talked about the similarities between the NA scope of practice and that of the RN, often struggling to differentiate between the two. For example, one trainee predicted at the start of their training that the roles would feel very similar in their clinical setting, which they confirmed in their third interview:

Basically, what we do in theatres is, as a trainee NA and as a NA, we will be doing the same role as what a RN does. So, we will eventually scrub for minor cases, and, as we're learning, we'll probably move up to bigger cases. TNA1 (yr 1)

I know there are some differences between a RN and a NA in different areas in the hospital. In theatres it's pretty much the same. The only thing that we wouldn't do in theatres would be you wouldn't be a team leader for the day. TNA1 (yr3)

This is another example of where a participant's initial concerns are supported by their experience over time. In support of this, another participant had read guidance on the differences in scope of practice between an NA and an RN. However, in practice, over time, they realised that there was an expectation to move beyond those boundaries.

Basically, we do exactly the same as qualified RNs do. There's not a lot of difference apart from IV medications and co‐ordinating the ward really. TNA17 (yr 2)

There's a template on the NMC [website] that says RN on one side, NA on the other, and it says the things that we can do and things that we can't do, but I mean, you can merge the line slightly… Basically, for me, I feel that the only difference between being an RN and being a NA, is that you can't give IVs in our trust. But I've read on a lot of forums that a lot of NAs are now giving IVs, and I think they're crazy, I think they're absolutely insane. TNA17 (yr3)

Initially, NAs viewed their identity as sitting between the HCA and RN roles. However, it became apparent through clinical experience that, in some settings, the role was closer to that of the RN.

#### 5.3.2 Colleagues' responses to the NA role

Most participants felt the NA role was misunderstood by their colleagues throughout their training journey, with some experiencing little understanding of the boundaries of the scope of NA practice. The following example illustrates little change in how the role is perceived by others over the duration of their training and as they transition into the workplace:

I don't think enough people actually know what a trainee NA is, maybe once it's been about for a bit, people will start to grasp it… I've just stopped saying that I'm a trainee NA anymore. When I do say it, I say, I'm just like the SEN [state enrolled nurse] you had years ago, because that's what they understand… you just have to adapt to what they understand, because they don't know what you can and what you can't do. TNA1 (yr2)

Unfortunately, because it's still such a new role, not everyone understands it. The clinical education team are amazing and they have a grasp and understand it. But…other teams; they don't really understand it fully yet, which is a shame, but hopefully as more go on the course and progress, then they'll understand.' TNA1 (yr3)

The vocational status of trainees, the fact they spent the majority of time working as employees, also meant colleagues often failed to recognise their student status. This had a direct impact on whether they were encouraged and facilitated to access learning opportunities which they felt were more often given to RN trainees who had a clearer student identity:

They'll spend time with [RN] student nurses, and show them things, but they won't spend time with you […] if you're on with [RN] student nurses, you can guarantee if there's a job to be done, or something to be learned, it won't be you that's picked. TNA6 (yr3)

Perhaps linked to this, some participants said that they were underutilised in their role being viewed more as HCAs than NAs by their RN colleagues. One talked about this in their first and second interviews, with no evidence that this perception had changed over time:

Because of the attitude of quite a high percentage of the RNs, they don't understand the role, and therefore, they don't treat us with any, well, respect really. We're just…we're treated as HCAs, but know a bit more than the other HCAs. TNA6 (yr 1)

A lot of the RN staff seem to think that this is just a slight upgrade from an HCA, as opposed to where we should be. They just seem to think it's a little bit of a step up from a HCA. It's like, yeah, you're an HCA that's done a bit of extra training rather than, you're a NA. TNA6 (yr2)

There was suggestion that some RNs were unsupportive of the role due to a reluctance to embrace change and that this influenced the offer of learning opportunities to trainee NAs:

I think some members of the team still actively resist the role, but they do it behind nursing rationale, when it doesn't need to be. I think nursing has its own language. And I think if you can speak it you get on a lot better, whereas if you can't… I think a lot of the RNs feel put out, a lot of them have been in the job a very long time. There's an in‐depth opposition to change. TNA8 (yr2)

In contrast to those feeling they were being underutilised, others felt they were expected to work beyond their scope of practice. This is evident in the clinical roles they were expected to perform, such as administering insulin to unstable people with diabetes or prescribing antibiotics:

On the wards, I think there is going to be an aspect of yes, you're an NA, but we want you to do extras, because I'm seeing it already, we want you to be doing more than you're supposed to be doing. TNA5 (yr1)

It's become a difficult thing to stand your ground when they are saying, ‘just go in, it's fine, they're fine’, and you say, ‘but they've had two hypos [hypoglycaemic episodes] in the last week, so I don't feel they're a stable patient’. TNA6 (yr3)

The persistence of this lack of understanding of the NA scope of practice over time is illustrated in the following extracts from one participant's interactions with medical colleagues, discussed in year 2 and 3 interviews:

People say to me… ‘oh you're a nurse now’, I'm like ‘no, no, I'm a Nursing Associate’. They're like, ‘well what's the difference’ …The doctors are like, ‘well why can't you give it’ and I'm like, ‘because I can't. I can't assess the patient, so I can't just go around just giving jabs here, there, and everywhere’. TNA16 (yr2)

The lack of understanding from GPs that I am not a RN. I'm a Nursing Associate and I have limitations to my scope of practice… I will often say to the GPs, ‘no, I can't do that, please don't put that in with me’… For example with a wound or something like that, a doctor will say ‘oh just prescribe antibiotics’… That's the sort of thing when I'm a bit like, ‘no, no, no that's your job, not my job’. TNA16 (yr3)

Participants were not passive in relation to such responses from colleagues though. Many developed strategies to help improve role clarity and thereby improve learning and development opportunities and the subsequent development of professional identity.

#### 5.3.3 Strategies to improve role clarity

Some participants described how they responded to misunderstandings about their scope of practice, by sign‐posting colleagues to online guidance or explaining what competencies they are able to undertake. This required self‐confidence and excellent communication skills. Educating colleagues enabled some participants to expand their scope to that of an NA:

It's been a challenge breaking down the barriers towards this role, because they just didn't understand what they could give me. So at first they were trying to send me out on visits which, you're basically just a carer… So I sort of did that for a little while and I thought, I'm not happy with this, there's so much more I can be doing. I then started to kind of push because I thought, I can do all this, I'm competent, wound care is my thing, bloods is my thing’ TNA12 (yr3)

One talked in her diary entry about a presentation on the NA role that she sends to people who want to know more, and another talked about re‐educating colleagues:

I always try to be proactive and positive about the role, so much so I have made my own short presentation that states our role very clearly. If anyone asks me ‘so what is a Nursing Associate’, I offer to send them my little PowerPoint. TNA12 (diary)

I've done a bank shift back on my old psych ward, and instantly I met the barriers that I had encountered previously by the colleagues there. And I was like, ‘no, I can do this, I can do that – and I actually can do a lot more than what I was doing when I was here previously’. And it was having to re‐educate some of the older RNs, to my role and my new autonomy. TNA13 (yr2)

Perhaps due to initiatives like these, one participant noted an improvement in how the role was understood over time, which helped enhance communication within the healthcare team:

I'm often going in and bringing up the proficiencies for NAs and bringing those up, which are on their website….The reception team have got a lot more understanding of it now as well. They've started to learn the difference between a HCA and an NA, which has been really good. They know who to approach now as well. So it's getting there. TNA16 (yr3)

In mitigating role ambiguity, some participants talked about initiatives which could be, or were being, introduced, such as trust‐wide education. However, there was some scepticism about how helpful this would be in the long term due to the flexibility within the guidance.

It might be a really good idea for there to be some sort of training…I think that for nursing staff, it might be of benefit to have a training afternoon, teaching them what we can do. TNA6 (yr3)

Trusts are now doing a lot more webinars, and getting in people from the NMC, HEE, …and so they did a six week series on NAs that was designed for anybody that wants to become a NA, any managers that have got NAs, or anybody that's thinking of hiring NAs. And the conclusion was, you can do what you want with the NA as long as there's clinical guidance to support that, but we don't know what you want to do with them so we can't write any, so you decide what you want to do with it and you write it. TNA2 (yr3)

Self‐perception and the responses of others had a clear part to play in building the NA professional identity. Several participants described the lack of role clarity, both in terms of their own experiences and the perceptions of those around them. They outlined strategies used to mitigate the barriers they encountered caused by misunderstandings of their scope of practice and place in healthcare teams.

## 6 DISCUSSION

The findings from this longitudinal study of NAs as they transitioned from training into practice reveal how they developed key attributes which contributed to building their professional identity (Fitzgerald, 2020). They illustrate the impact of personal and organisational understanding of the role, in addition to the acquisition of knowledge skills and an increased sense of responsibility for patient care. This journey was not without challenges. Similarly, in a review of workplace transitions, Arrowsmith et al. (2016) describe the discomfort experienced by those striving for a new identity.

### 6.1 Development of knowledge, skills and responsibility

Participants described how their skills and knowledge developed over time, enabling them to provide an enhanced level of care for patients compared to their previous healthcare support roles. Similarly, Maginnis (2018) reflects on the importance of developing skills and knowledge through both academic and quality clinical placements in contributing to the building of professional identity. The ‘four fields’ nature of the NA training programme enabled them to care for patients across a range of settings and helped distinguish them further from RNs. RNs undertake specific training in adult, child, learning disability or mental health nursing, whereas the NA programme equips trainees to fulfil a generic role across all of these fields post‐qualification (NMC, 2018a, 2018b). This gave some increased confidence in their clinical decision‐making and interactions with colleagues. Similarly, in their qualitative study of student nurses Fitzgerald and Clukey (2021) found that professional identity was enhanced through increased confidence in decision‐making; developed through stronger competence. Previous research has identified skills acquisition as an important step in developing competent practice (Arrowsmith et al., 2016).

Professional regulation and registration with the NMC provided a further sense of identity, with a recognition by participants of the responsibility and accountability that registration brings. Those who had previously worked as HCAs reflected on the move from delivering task‐based care to becoming person‐centred decision‐makers.

### 6.2 Perceptions of role identity

Professional identity was also moulded through the self‐perception of the role by NAs and their experiences of the views of their colleagues. This is consistent with research on transitions, where nurses look to their colleagues as a reference when forming a new identity (Arrowsmith et al., 2016). The current findings revealed that NAs perceived their role to be very similar to that of RNs, leading to frustrations; a common experience in situations of boundary blurring (Fitzgerald, 2020; Nancarrow & Borthwick, 2005). Although the role of RN and NA are distinguished in regulatory standards (NMC, 2018a, 2018b), this study indicates that the NA role is sometimes stretched beyond these boundaries to meet service needs. Similarly, in a study of the nurse–medical boundary, Johannessen (2018) reflected that blurring of workplace boundaries was not always reflected in formal jurisdictions.

Boundary blurring between nurses and doctors was perceived by Allen (1997) as an inevitable consequence of the absence of doctors in clinical practice (Allen, 1997), therefore it is not surprising to see this being played out between NA and RN roles as the nursing workforce faces unprecedented shortages (WHO, 2022). In the present study, there are times when the colleagues of NAs expected them to work beyond their scope of practice. This boundary blurring caused tensions among participants; it could leave them feeling out of their depth and believing they did not receive adequate remuneration for their work. Previous research has shown that this frustration can lead some NAs to pursue further training to become RNs, a mechanism by which they can gain formal jurisdiction for their work (King et al., 2022).

A lack of role clarity among colleagues led some participants to feel underutilised in their role, while others said that too much was expected of them. In their study of the implementation of the advanced practice role, Schober et al. (2016) found that role clarity was crucial, as ambiguity led to isolation and a lack of acceptance by other healthcare professionals. However, over time, inter‐professional resistance decreased as understanding of the role increased. Similarly, Rees et al. (2019) explored professional identity within interprofessional teams, finding both a persisting narrative of interprofessional conflict, in addition to one of interprofessional collaboration. They argue for work‐based training to embed both professional and inter‐professional identities in students and clinicians. Furthermore, Arrowsmith et al. (2016) argue that managers must ensure clarity of role boundaries in role transitions. Concerns about the impact of role ambiguity around second‐level nursing (Lucas et al., 2021) have persisted in the NA role. At a time when the nursing workforce is experiencing a workforce and economic crisis (WHO, 2022), healthcare teams should pull together to support each other. Strategies to mitigate role ambiguity and enhance retention through valuing the work of this new member of the team are essential.

Concerns have been raised by the RCN, (2023) about how role confusion could lead to risks to NAs, who may act outside of their regulatory framework. In order for NAs to avoid the persisting ambiguity experienced by second‐level nurses internationally (Lucas et al., 2021), there needs to be strong investment in clarifying the scope of practice and recognising role boundaries. NA role models who are clear about their place and scope of practice in healthcare teams will be crucial in establishing a professional identity in the future. They could benefit from communities of practice of NAs (Lave & Wenger, 1991) as a mechanism by which to strengthen their professional identity across England. The successful negotiation of a professional identity for NAs requires all stakeholders to champion the role and to educate the wider health and social care teams on the scope of practice of NAs in differing health and social care settings.

### 6.3 Strengths and limitations of the work

The strength of this study design lies in the longitudinal nature of data collection and the subsequent rapport developed between the researchers and participants (Neale, 2020). Also, the timing of this study has been valuable in exploring the concept of professional identity building at the early stages of NA role implementation across England.

This study included a small sample size, however, due to the longitudinal nature of qualitative data collection, a sufficient depth of understanding of NA professional identity formation was achieved. There was a small loss of participants across the 3 years but most continued to take part suggesting good engagement with the study. Although the sample achieved diversity of age and geography, most of the participants were female and white British, future research should therefore aim for greater diversity of ethnicity and gender.

### 6.4 Recommendations for further research

It is important to explore how the scope of the role is enacted in a range of health and social care settings to further understand the professional identity of NAs. Future research should explore the work of NAs in practice to understand the extent to which there is substitution of RNs by nursing associates and the impact this has on patient safety.

As the NA role becomes more established it would be helpful to study the nature of communities of practice and role models in supporting trainees and newly qualified NAs of the future. At a time when recruitment and retention of healthcare workers is paramount, future research should evaluate interventions to support the career development and well‐being of NAs and similar second‐level nurses internationally.

## 7 CONCLUSION

The professional identity of newly qualified NAs is developed through the acquisition of knowledge and skills, increased responsibility and the perceptions of the role by NAs and their colleagues. Regulation by the NMC professional body enhances professional identity by increasing a sense of responsibility through accountability.

NAs in this study clearly valued their new knowledge, skills and responsibility for patient care. Tensions arose when the scope of NA practice expected by organisations was over or under that expected by the NA. Frustrations also occurred when NAs felt their role was significantly blurred with that of RNs as they did not want to be perceived as substitutes for RNs in the context of nursing workforce shortages. National guidance and employers should provide clarity of the boundaries of the NA role which reflect the differences between the NA and RN roles to strengthen the professional identity of NAs and mitigate role ambiguity within health and social care organisations.

## AUTHOR CONTRIBUTIONS

Rachel King: Conceptualisation, data curation, formal analysis, investigation, project administration and writing original draft. Sara Laker: Conceptualisation, data curation, formal analysis and writing original draft. Bethany Taylor: Conceptualisation, data curation, formal analysis, investigation and writing review and editing. Tony Ryan: Conceptualisation, funding acquisition, supervision and writing review and editing. Emily Wood: Conceptualisation, methodology and writing review and editing. Angela Tod: Conceptualisation, funding acquisition, methodology, supervision and writing review and editing. Michaela Senek: Conceptualisation, methodology and writing review and editing. Sally Snowden: Conceptualisation, resources and writing review and editing. Steve Robertson: Conceptualisation, data curation, formal analysis, investigation, methodology and writing original draft.

## FUNDING INFORMATION

The project was carried out by the Strategic Research Alliance between The Royal College of Nursing and the University of Sheffield. The views expressed are those of the author(s), and not necessarily those of the RCN.

## CONFLICT OF INTEREST STATEMENT

There are no conflicts of interest.

## Data Availability

The data that support the findings of this study are available from the corresponding author upon reasonable request.
